# The Novel Progressive Ratio with Reset Task Reveals Adaptive Effort-Delay Trade-Offs

**DOI:** 10.1523/ENEURO.0467-25.2026

**Published:** 2026-01-30

**Authors:** Gayle A. Edelstein

**Affiliations:** Behavioral Neuroscience Program, Department of Psychological Sciences, University of Connecticut, Storrs, Connecticut 06269-1020

The progressive ratio (PR) schedule is a popular and well-established tool used to study decision-making and effort across species. In this task, subjects perform an instrumental response to receive a reinforcement, but the ratio requirement increases throughout the behavioral session. A subject's breakpoint, or the point at which the subject is no longer willing to exert the effort required to receive reinforcement, is the main behavioral readout analyzed in the PR schedule. The PR schedule has been used to investigate motivation for various reinforcers (e.g., drugs of abuse) and has been adapted to incorporate aspects of effort-related decision-making (e.g., choice between high- and low-value outcomes). However, there are notable limitations to the utility of the PR as a test of decision-making, including the confounding effects of effort and delay in that higher ratios take longer to complete, the limited behavioral response options (e.g., lever pressing), and breakpoint being a low-dimensional measurement that lacks sensitivity.

To address these limitations, Rivera et al. (2025) developed a variation of the PR that incorporates a strategically meaningful opportunity for rats to control the effort requirement throughout the task. PR with reset (PRR) tasks provide rats with the opportunity to press a second lever at any point within the session to reset the ratio requirement back to one, but at the cost of a delay during which no reinforcers can be earned. The addition of the ratio option segments behavior into discrete bouts of work. Each bout then begins with a low-effort, high-reward phase and ends with the choice of continuing to lever press at a high ratio or pressing the reset lever and incurring the cost of a delay. While previous studies have investigated the utility of having a cost-free reset option on the PR schedule (Hurwitz and Harzem, 1968), the PRR method alters the economic structure. Now, subjects must find an optimal behavioral strategy that limits exertion of effort without resetting the ratio too frequently as the delay would consume a large fraction of the behavioral session. The PRR task taps into a wider range of behavioral strategies used for cost–benefit decision-making as compared with the PR schedule.

Rivera et al. (2025) developed and assessed two different versions of the PRR task, the PRR-10 which has a 10 s reset delay, and a PRR-60 which has a 60 s reset delay. Male and female Long–Evans rats were tested on a random sequence of PR, PRR-10, and PRR-60 sessions. To evaluate rats’ choices on these tasks, a normative modeling framework grounded in foraging theory, due to the similarity in tasks and behavioral output, was applied (Stephens and Krebs, 1986).

Characterization of behavior on the PR versus the PRR tasks revealed important variations. Rats earned the most rewards with each bout of work on the PRR-60 compared with PRR-10 sessions, but the total average earnings were greatest for the PRR-10. Bout length, total active presses, and active presses per session were highest in the PR and did not differ between PRR tasks. While reward collection latency did not differ between tasks, return to work latency did with greater latency on the PR versus PRR-10 and PRR-60. Interestingly, female rats took ∼2 s longer on average to resume working toward completing the next ratio compared with males. Although generally used sparingly throughout all three tasks, the use of the reset lever occurred most frequently in PR where it imposed no consequences, but its use decreased in frequency with increase in reset delay.

Analysis of reset lever use on the PRR tasks found that reset latency with PRR-60 peaked later (∼40 s) compared with PRR-10 (∼25 s), indicating that rats spent substantial time earning rewards between reset presses when the reset delay was higher and more costly. Bout length was affected by reset delay as the PRR-10 produced the shortest bout length compared with the PRR-60 and PR. The reset delay increased bout length, but not total number of bouts, more strongly in male rats compared with females.

The structure of the PRR tasks mimic features of the patch-leaving foraging problems, in which animals harvest diminishing resources in their current patch and have the choice to relocate at a cost (MacArthur and Pianka, 1966). Since optimality modeling is frequently used in patch-foraging scenarios (Emlen, 1968), Rivera et al. (2025) sought to apply the same approach to the PRR tasks to determine the optimal behavioral strategy to maximize net energy.

Using active-lever-press rate and reset-delay length, an optimal bout length for each PRR session was measured and used as the optimal strategy to compare to rats’ behavior. Observed bout length was strongly correlated with optimal bout length in each PRR session meaning that the rats’ behavior matched appropriately to the rate of lever pressing maintained in each session. However, rats were systematically biased toward longer-than-optimal bout lengths, reminiscent of the “overharvesting” observed on patch-leaving tasks (Nonacs, 2001). Further analyses found that sex and active-lever-press rate predicted the tendency of rats to complete “excess” ratios per bout of work. While male rats had a greater number of excess ratios per bout of work than females, active press rate, but not reset delay duration, was a significant predictor of excess ratios in both males and females.

The PRR tasks tap into a similar form of decision-making as standard PR but with a more naturalistic decision topology. Compared with standard PR, the PRR tasks provide improved sensitivity and result in more fine-grained features of behavior. PRR results allow for the dissociation of the effects of temporal preferences and effort sensitivity using a single behavior. This provides an avenue for research related to preclinical features of various neuropsychiatric disorders associated with alterations in time sensitivity and effort costs as well as assessment of neural mechanisms underlying decision-making.
